# Barriers and Opportunities of Oncofertility Practice in Nine Developing Countries and the Emerging Oncofertility Professional Engagement Network

**DOI:** 10.1200/JGO.18.00180

**Published:** 2018-12-27

**Authors:** Mahmoud Salama, Lauren Ataman-Millhouse, Fabio Sobral, Guillermo Terrado, Anibal Scarella, Maria T. Bourlon, Satish Kumar Adiga, Karthik S. Udupa, Nalini Mahajan, Madhuri Patil, Chris Venter, Georgia Demetriou, Ramiro Quintana, Gabriela Rodriguez, Tomas Quintana, Luz Viale, Yuly Andrea Remolina Bonilla, July Andrea Russi Noguera, Juan Carlos Velásquez Velásquez, Jennifer Ivonne Dominguez Pineda, Mario Daniel Castro Aldecoa, Murid Javed, Hamad Al Sufyan, Nonso Daniels, Adegbite A. Ogunmokun, Teresa K. Woodruff

**Affiliations:** ^1^ Northwestern University, Chicago, IL; ^2^ National Research Center, Cairo, Egypt; ^3^ Pregna Medicina Reproductiva, Buenos Aires, Argentina; ^4^ Universidad de Valparaiso, Valparaiso, Chile; ^5^ Instituto Nacional de Ciencias Médicas y Nutrición Salvador Zubirán, Mexico City, Mexico; ^6^ Manipal Academy of Higher Education, Manipal, India; ^7^ Mother and Child Hospital, New Delhi, India; ^8^ Private Fertility and Endoscopy Clinic, Bangalore, India; ^9^ Vitalab Fertility Centre, Johannesburg, South Africa; ^10^ University of Witwatersrand, Johannesburg, South Africa; ^11^ Procrearte, Buenos Aires, Argentina; ^12^ Instituto Nacional de Cancerología, Bogota, Colombia; ^13^ Instituto Guatemalteco de Seguridad Social, Guatemala City, Guatemala; ^14^ Thuriah Medical Center, Riyadh, Saudi Arabia; ^15^ The Oncology and Fertility Centres of Ekocorp, Eko Hospitals, Lagos, Nigeria

## Abstract

**PURPOSE:**

Oncofertility practice continues to grow in developing countries despite the lack of health care services, especially those related to cancer care. The purpose of this study is to further explore oncofertility practice in these countries and identify opportunities for field-wide coalescence.

**METHODS:**

We generated a survey to learn more about oncofertility practice in nine developing countries within our Oncofertility Consortium Global Partners Network—Mexico, Colombia, Guatemala, Argentina, Chile, Nigeria, South Africa, Saudi Arabia, and India. Their responses were collected, reviewed, and discussed.

**RESULTS:**

Surveyed centers from the nine developing countries continue to experience a similar set of common challenges, including a lack of awareness among providers and patients, cultural and religious constraints, lack of insurance coverage and funding to help to support oncofertility programs, and high out-of-pocket costs for patients. Despite these barriers, many opportunities exist and there is great potential for the future.

**CONCLUSION:**

The current need is to unify the new technologies and best practices that emerge from rural communities and developing countries with those in large metropolitan cities, both domestically (US based) and abroad, into a functional unit: the Oncofertility Professional Engagement Network. The Oncofertility Professional Engagement Network will bridge the gap between domestic and international programs to establish a strong global network in which members share resources, methodologies and experiences and further build cultural competency.

## INTRODUCTION

The Oncofertility Consortium Global Partners Network (OCGPN), established at the Feinberg School of Medicine, Northwestern University, Chicago, IL, aims to provide edification and modeling to oncofertility providers around the globe, especially in developing countries that lack several health services related to cancer care.^1-3^ Limited resources in the developing countries makes their proper allocation and exploitation of the utmost necessity, particularly in a new and complex medical field, such as oncofertility. Recently, OCGPN has published a pilot survey concerning oncofertility practice in five developing countries—Egypt, Tunisia, Brazil, Peru, and Panama.^4^ The study concluded that, despite barriers to care, many opportunities exist to grow the field of oncofertility in these five developing countries. The study also encouraged engaging stakeholders in developing countries and using powerful networks in the United States and other developed countries to aid in the acceptance of oncofertility on a global level.^4^ As a consequent step, OCGPN has expanded the oncofertility survey and involved in the current study nine developing countries from Latin America, Africa, and Asia—Mexico, Colombia, Guatemala, Argentina, Chile, Nigeria, South Africa, Saudi Arabia, and India—to help them investigate their own barriers and highlight their own opportunities.

## METHODS

Survey questions were sent by e-mail to nine centers from Latin America, Africa, and Asia within the OCGPN. Surveyed centers from Mexico, Colombia, Guatemala, Argentina, Chile, Nigeria, South Africa, Saudi Arabia, and India are listed in Appendix Table A1. Survey questions were grouped into six categories: country profile, cancer care, fertility treatments, fertility preservation treatments, barriers to oncofertility, and opportunities of oncofertility (Data Supplement). Responses from surveyed centers were collected, reviewed, and discussed.

## RESULTS

All surveyed centers from the nine developing countries—Mexico, Colombia, Guatemala, Argentina, Chile, Nigeria, South Africa, Saudi Arabia, and India—responded to all questions. Responses are listed in detail in the Data Supplement—developing country profile 2016 and 2017, cancer care, fertility treatments, fertility preservation treatments, barriers to oncofertility, and opportunities of oncofertility.

## DISCUSSION

According to the United Nations Human Development Reports 2016 and 2017,^5^ most developing countries included in this survey have lower-to-upper-middle income economies with a low public health expenditure as a percentage of gross domestic product (less than 4%). State health insurance is still developing and does not cover the majority of the population in lower-middle-income countries, such as India, South Africa, and Nigeria. Of interest, Nigeria showed the highest fertility rates (5.59 births per woman) and the lowest life expectancy (age 56 years for women and 53 years for men; Data Supplement).

According to the WHO GLOBOCAN Study 2012,^6^ most developing countries showed lower cancer incidence rates compared with developed countries because of a lack of national programs for screening, diagnosis, and registration. Surveyed centers reported that the most common cancers among women are breast, cervical, uterine, lung, colorectal, and stomach, whereas the most common cancers among men are lung, liver, stomach, prostate, and colorectal. Most cancer treatments are provided for free or are covered by insurance. Cancer treatment providers include national cancer institutes, university hospitals, specialized cancer hospitals, and public hospitals, and all of which provide services that are either free or covered by insurance. Some major private hospitals provide cancer treatments that are covered by insurance or out-of-pocket payment. Despite of the growing attention to the disease, cancer prevention and treatment services are still not sufficient, and the official national registries are still under development in some countries, such as Mexico, Guatemala, and Nigeria (Data Supplement).

As a result of cultural reasons, most developing countries have high fertility rates, as in Nigeria (5.59 births per woman). In the case of infertility, patients seek treatments early to avoid future social pressure. In Saudi Arabia, Nigeria, and India, fertility treatments are provided only to married heterosexual couples because of conservative cultural and religious reasons. In most countries, the following assisted reproductive techniques are available: intrauterine insemination, in vitro fertilization and intracytoplasmic sperm injection, and cryopreservation of sperm, embryo, and oocytes. Third-party reproduction is unregulated or prohibited in most countries as a result of conservative cultural and religious reasons. The majority of fertility services are provided in private centers and are not covered by insurance. Some public centers at university hospitals may offer low-cost fertility services and some charities may support patients with limited resources. The average cost of a single cycle of in vitro fertilization and intracytoplasmic sperm injection is widely variable, starting from 1,500 USD in India and reaching 10,000 USD in Chile. Success rates of fertility treatments seem to be comparable to international standards, although official national registries are still missing in some countries, such as Mexico, Colombia, Guatemala, Nigeria, and Saudi Arabia (Data Supplement).

In most countries that participated in this survey, fertility preservation treatments are provided mainly to patients without cancer during assisted reproductive technique treatments to cryopreserve embryos, sperm, or oocytes. Unfortunately, patients with cancer are usually not informed about the available fertility preservation options because of a lack of awareness among providers. Available fertility preservation treatments are cryopreservation of sperm, embryo, and oocytes; however, in vitro maturation, ovarian tissue freezing, and testicular tissue freezing are not yet available in most countries as a result of a lack of technology and trained teams. Social egg freezing is still uncommon because of a lack of awareness. Success rates of fertility preservation treatments in most countries are still below international standards and no national registry for fertility preservation services is available in any country included in this survey (Data Supplement).

There are several common medical, economic, social, and legal barriers to oncofertility practice in the surveyed countries. Medical barriers include a lack of awareness among oncologists and gynecologists, lack of advances in early diagnosis and treatment of cancer, low referrals from oncologists, deficient interinstitutional communication, and the absence of oncofertility specialists. Economic barriers include the lack of health insurance coverage for fertility services, lack of institution and research funding, and exclusively high costs; a majority of fertility services are provided in private centers and paid as out-of-pocket services. All of these factors create a financial burden to patients. Social and legal barriers include conservative religious, cultural, and ethical attitudes that prohibit third-party reproduction in some countries (Data Supplement).

Despite different barriers, oncofertility still has great potential in the surveyed countries for the following reasons: Fertility preservation is the most suitable way for patients with cancer to have children, especially in countries with conservative culture and high fertility rates; cryopreservation of sperm, embryo, and oocytes is already available; cancer diagnosis and treatments are improving at new cancer hospitals; spreading awareness among oncologists, gynecologists, and patients can be achieved via oncofertility networks, media, and repeated promotion campaigns; and financial support for patients, technology, training, and research can be achieved via national and international grants, charities, and fundraising campaigns (Data Supplement).

**FIG 1:**
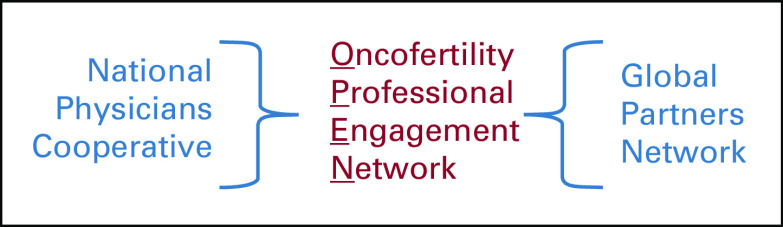
Merger of domestic and global networks in to one unified network, the Oncofertility Professional Engagement Network.

Our survey confirmed that barriers to oncofertility care still exist in developing countries with limited resources; however, it is also clear that there is momentum for clinical and translational oncofertility activities worldwide, as confirmed by several international guidelines.^7-24^ As a testament to the success of the OCGPN, the network continues to grow in terms of the number of participating centers, which can be equated to an increase in the number of patients reached worldwide. Through our efforts, the OCGPN now spans six continents, including more than 40 countries around the globe and 85 sites in the United States. After a thoughtful evaluation of the status of the field and the evolving needs of its members, it became clear that as the number of participating centers increases, there is no longer a need to separate these centers geographically (US based *v* international). We now aim to move toward the coalescence of the individual stakeholders in the field to OPEN (Fig 1). As the field consists of a vast network of diverse individuals from around the globe, it is important that members see themselves as oncofertility ambassadors. This inclusiveness improves the performance, skills, and attitudes of oncofertility stakeholders. In addition, working together collectively can help to highlight the importance on this field in patient care to be considered in the future as part of the public budget in developing countries. OPEN helps us move forward from a previous model that separated networks on the basis of geography—domestic versus global—toward an inclusive model that allows all stakeholders to participate in the same activities regardless of physical location. We have connected common goals and interests to enable a network of engaged professionals and trainees, both in the United States and abroad, who share a passion for oncofertility and reproductive health.^25^ These teams have created a series of intellectual and didactic products, but until now this work has largely been segregated within domestic and international sites.^1-4^ To capture the full intellectual capacity of the group, OPEN will create a framework by which the entire field can first share information, then translate it to fit the individual needs of each unique center, thereby transforming the field into a globally recognized, yet culturally sensitive, field.^26^

In conclusion, the surveyed centers from the nine developing countries continue to experience a similar set of common challenges, including a lack of awareness among providers and patients, cultural and religious constraints, lack of insurance coverage and funding to help support oncofertility programs, and high out-of-pocket costs for patients. The current need is to unify the new technologies and best practices that emerge from rural communities and developing countries with those in large metropolitan cities, both domestically (US based) and abroad, into a functional unit, OPEN. OPEN will bridge the gap between domestic and international programs to establish a strong global network in which members share resources, methodologies, and experiences, and further build cultural competency in the field of oncofertility.

## Figures and Tables

**TABLE A1:**
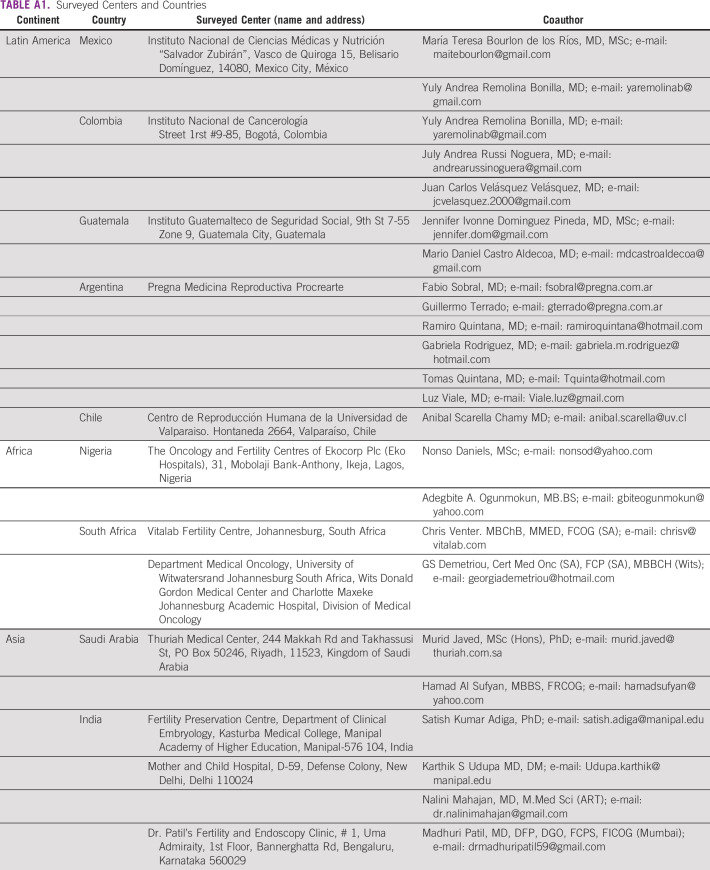
Surveyed Centers and Countries

## References

[1] Ataman LM Rodrigues JK Marinho RM et al: Creating a global community of practice for oncofertility. J Glob Oncol doi:10.1200/JGO.2015.000307 10.1200/JGO.2015.000307 PMC4894337 27284576

[2] Rashedi A de Roo SF Ataman L et al: A survey of fertility preservation options available to cancer patients around the globe. J Glob Oncol doi:10.1200/JGO.2016.008144 10.1200/JGO.2016.008144 PMC7853877 32259160

[3] Rashedi A de Roo SF Ataman L et al: A survey of third-party parenting options associated with fertility preservation available to patients with cancer around the globe. J Glob Oncol doi:10.1200/JGO.2017.009944 10.1200/JGO.2017.009944 PMC7853875 32259159

[4] Salama M Ataman L Taha T et al: Building oncofertility core competency in developing countries: Experience from Egypt, Tunisia, Brazil, Peru, and Panama. J Glob Oncol doi:10.1200/JGO.17.00121 10.1200/JGO.17.00121 PMC7853873 32259156

[5] United Nations: Human development reports: Global human development indicators. http://hdr.undp.org/en/countries

[6] International Agency for Research on Cancer: Population fact sheets. http://globocan.iarc.fr/Pages/fact_sheets_population.aspx

[7] Lee SJ Schover LR Partridge AH et al: American Society of Clinical Oncology recommendations on fertility preservation in cancer patients. J Clin Oncol 24:2917-2931 2006 16651642 10.1200/JCO.2006.06.5888

[8] Loren AW Mangu PB Beck LN et al: Fertility preservation for patients with cancer: American Society of Clinical Oncology clinical practice guideline update. J Clin Oncol 31:2500-2510 2013 23715580 10.1200/JCO.2013.49.2678 PMC5321083

[9] Oktay K Harvey BE Partridge AH et al: Fertility preservation in patients with cancer: ASCO Clinical Practice Guideline Update. J Clin Oncol 36:1994-2001 2018 29620997 10.1200/JCO.2018.78.1914

[10] Ethics Committee of American Society for Reproductive Medicine: Fertility preservation and reproduction in patients facing gonadotoxic therapies: A committee opinion. Fertil Steril 100:1224-1231 2013 24094423 10.1016/j.fertnstert.2013.08.041

[11] Practice Committee of American Society for Reproductive Medicine: Fertility preservation in patients undergoing gonadotoxic therapy or gonadectomy: A committee opinion. Fertil Steril 100:1214-1223 2013 24011612 10.1016/j.fertnstert.2013.08.012

[12] Pentheroudakis G Orecchia R Hoekstra HJ et al: Cancer, fertility and pregnancy: ESMO Clinical Practice Guidelines for diagnosis, treatment and follow-up. Ann Oncol 21:v266-v273 2010 (suppl 5) 20555095 10.1093/annonc/mdq198

[13] Peccatori FA Azim HA Jr Orecchia R et al: Cancer, pregnancy and fertility: ESMO Clinical Practice Guidelines for diagnosis, treatment and follow-up. Ann Oncol 24:vi160-vi170 2013 (suppl 6) 23813932 10.1093/annonc/mdt199

[14] Woodruff TK: The Oncofertility Consortium--addressing fertility in young people with cancer. Nat Rev Clin Oncol 7:466-475 2010 20498666 10.1038/nrclinonc.2010.81 PMC3124936

[15] Waimey KE Duncan FE Su HI et al: Future directions in oncofertility and fertility preservation: A report from the 2011 Oncofertility Consortium Conference. J Adolesc Young Adult Oncol 2:25-30 2013 23610740 10.1089/jayao.2012.0035 PMC3604786

[16] Kim SS Donnez J Barri P et al: Recommendations for fertility preservation in patients with lymphoma, leukemia, and breast cancer. J Assist Reprod Genet 29:465-468 2012 22648282 10.1007/s10815-012-9786-y PMC3370045

[17] Klemp JR Kim SS; ISFP Practice Committee: Fertility preservation in young women with breast cancer. J Assist Reprod Genet 29:469-472 2012 22614158 10.1007/s10815-012-9791-1 PMC3370052

[18] Jadoul P Kim SS; ISFP Practice Committee: Fertility considerations in young women with hematological malignancies. J Assist Reprod Genet 29:479-487 2012 22614159 10.1007/s10815-012-9792-0 PMC3370036

[19] Schmidt KT Andersen CY; ISFP Practice Committee: Recommendations for fertility preservation in patients with lymphomas. J Assist Reprod Genet 29:473-477 2012 22562284 10.1007/s10815-012-9787-x PMC3370035

[20] Coccia PF Pappo AS Beaupin L et al: Adolescent and young adult oncology, version 2.2018, NCCN Clinical Practice Guidelines in Oncology. J Natl Compr Canc Netw 16:66-97 2018 29295883 10.6004/jnccn.2018.0001

[21] Fallat ME Hutter J; American Academy of Pediatrics Committee on Bioethics et al: Preservation of fertility in pediatric and adolescent patients with cancer. Pediatrics 121:e1461-e1469 2008 18450888 10.1542/peds.2008-0593

[22] Fernbach A Lockart B Armus CL et al: Evidence-based recommendations for fertility preservation options for inclusion in treatment protocols for pediatric and adolescent patients diagnosed with cancer. J Pediatr Oncol Nurs 31:211-222 2014 24799444 10.1177/1043454214532025 PMC5213740

[23] Schüring AN Fehm T Behringer K et al: Practical recommendations for fertility preservation in women by the FertiPROTEKT network. Part I: Indications for fertility preservation. Arch Gynecol Obstet 297:241-255 2018 29177593 10.1007/s00404-017-4594-3 PMC5762797

[24] von Wolff M Germeyer A Liebenthron J et al: Practical recommendations for fertility preservation in women by the FertiPROTEKT network. Part II: Fertility preservation techniques. Arch Gynecol Obstet 297:257-267 2018 29181578 10.1007/s00404-017-4595-2 PMC5762782

[25] Lungeanu A Contractor NS: The effects of diversity and network ties on innovations: The emergence of a new scientific field. Am Behav Sci 59:548-564 2015 26576061 10.1177/0002764214556804 PMC4643280

[26] Northwestern University: The Oncofertility Consortium: Oncofertility Professional Engagement Network. http://oncofertility.northwestern.edu/oncofertility-professional-engagement-network

